# The influence of substitution decisions made by national team coaches on final match outcomes at UEFA EURO 2024

**DOI:** 10.3389/fspor.2025.1573823

**Published:** 2025-07-10

**Authors:** Tomasz Gabrys, Radoslaw Chruscinski, Michal Garnys, Matej Suva, Karel Švátora

**Affiliations:** ^1^ Sport Centrum Faculty of Education, University of West Bohemia, Pilsen, Czechia; ^2^ Department of Sport Science, 4Sport LAB, Warsaw, Poland; ^3^ Faculty of Health Science, University of Applied Science, Nysa, Poland

**Keywords:** football competition, player substitutions, coaching decision, machine learning, game strategy

## Abstract

**Introduction:**

Coaches leading national football teams during championship tournaments make decisions about tactical substitutions of players in critical match phases. This may be an attempt to change or defend a favorable score. This study focused on the time of decision-making of forced and planned substitutions, considering its characteristics: neutral, offensive, and defensive. The point of analysis of the substitutions was the match outcomes at the time of the substitutions and the final result and impact of the substitution concerning the result.

**Methods:**

A total of 51 matches played during the UEFA EURO 2024 football tournament were analyzed, during which 466 player substitutions were made. For the statistical analysis of the degree and strength of the relationship between the variables, the chi-square test, Cramer's V coefficient, and machine learning were used accordingly.

**Results:**

72% of coaches’ decisions to player substitutions resulted from the decision to change the team's tactics by changing the team's setup or the players’ positions. The most common negative (69%) or positive (61%) impact occurred from the substitution of a player after the 20th minute.

**Discussion:**

The decision trees used in the analysis determined the most advantageous time periods for coaches to make decisions about substitutions. The highest substitution effectiveness rate is obtained when the substitution is made between 60 and 85 min, and the lowest is made between 45 and 60 min.

## 1 Introduction

The basic skill of a football coach is the ability to manage a team in response to changing situations on the pitch (1). The goal is known; it is to win the match or, in some tournament situations, to secure a draw. Additional objectives that may result from the tournament table may be to score a certain number of goals or lose by a certain goal difference. Such a complex arrangement of the main goal and intermediate goals places very specific demands on the coach in terms of managing changing situations (2). The accuracy of the player substitutions determines the changes in the team's performance (3–5). An ineffective substitution is a mistake that is practically impossible to fix. Any substitution of a substituted player reduces the number of possible tactical options in the final part of the match (6, 7). Substitutions are primarily about rebuilding the team's physical potential (8). Decisions to make substitutions have a different nature. One of them is the changed efficiency of running performance. There are cases in which coaches consciously place players in the team who are not prepared to play the entire match. This situation applies to all positions except the goalkeeper, although more often to forwards and defenders. No studies have been found that take into account the player's lack of preparation to participate in the entire match and the resulting time of his substitution. The running performance of players is an indicator that influences their perception by the coach in the context of substitution. Some positions are more physically demanding, which forces more frequent use of substitutions (9, 10). Trewin (11) identifies the differences in the running strategy of players participating in the entire match and substitutes, showing that substitutes from the last 20 min of the match are characterized by a significantly higher work intensity (distance covered per minute of the match, distance with high intensity, accelerations). Substitutes may use all-out tactics more often, knowing that they will only play for a relatively short time in the match. However, Trewin's (11) research has limitations resulting from the sample size and requires verification on a wider group. During championship matches, especially tournaments, mitigating the effects of fatigue is the primary reason for making substitutions, in addition to changing the tactical solution (4, 5, 12). Tactical changes usually occur after the first 45 min of the match. Decreases in players' performance are visible throughout the match, but the most common breakdown occurs after approximately 60 min of the match (13–15). This is when the second peak in the number of substitutions (apart from the start of the second half) is visible (16). The third peak occurs around the 80th minute of the match when introducing a new player, which is usually aimed at increasing the intensity of the game and surprising the opponent with a change in the team's tactics (17–19). One of the criteria for an effective substitution is to achieve or exceed the intensity of the substituted players (13, 20), indicating that players introduced in the second half of the match performed 25%–63% more intensive running and sprinting in the last 15 min of the match compared with players who were on the pitch from the beginning of the match. In addition, Carling et al. (3) indicate that substitutes are characterized not only by relatively higher total distance and maximal intensity distance but also by shorter recovery time between repetitions. Bradley et al. (5) indicate that substitutes cover a greater relative distance in high-intensity running (19.8–25.1 km/h) than that in players who played the full match or were replaced. Strategic changes during the match's second half led to a reduced fatigue effect on the entire team. This observation was confirmed by the research of Hills et al. (12). Myers (16) formulates a decision rule according to which, if the team is losing, the change should be made before the 58th minute, the second before the 73rd minute, and the third before the 79th minute. The effectiveness of this rule is at the level of 42.27%, and the lack of its application in only 20.52% of cases allowed for a favorable change in the result. Considering many factors accompanying the course of the match accompanies the coach's decision-making process when deciding to make a change. The substitution has an impact on the team's tactical formation (17). An offensive substitution increases the probability of not only scoring a goal but also conceding one. The observation by Schneemann and Deutscher (21) indicates that when the team is losing, the intensity of the substitutes' effort is lower than expected. Therefore, the effectiveness of the substitution from the point of view of the result at which it occurs may be different, and an unfavorable result reduces the effectiveness of the substitution. In addition to the two basic tasks accompanying the change, i.e., maintaining or increasing the intensity of the team's play and correcting tactics, numerous studies indicate several other factors. Raab et al. (22) draw attention to the psychological aspect. The coach's decisions to make a change are also influenced by the experience of the player being changed (23, 24), the position of the host of the match (25–27), and the way of refereeing (28, 29). The tactical effect of changes may also have another dimension, not only strictly football-related, realized in the game. The influence of contextual factors on the coach's decisions cannot be ignored (18). These include the match's location, which increases the probability of modifying the initial formation and increased pressure from the fans (30), the current result of the match, and the losing team deciding to change earlier than the winning team (18). The quality of the opponent's game (31, 32) or the importance of the competition (33) also influences decisions about changes in the team composition.

**Figure 1:**
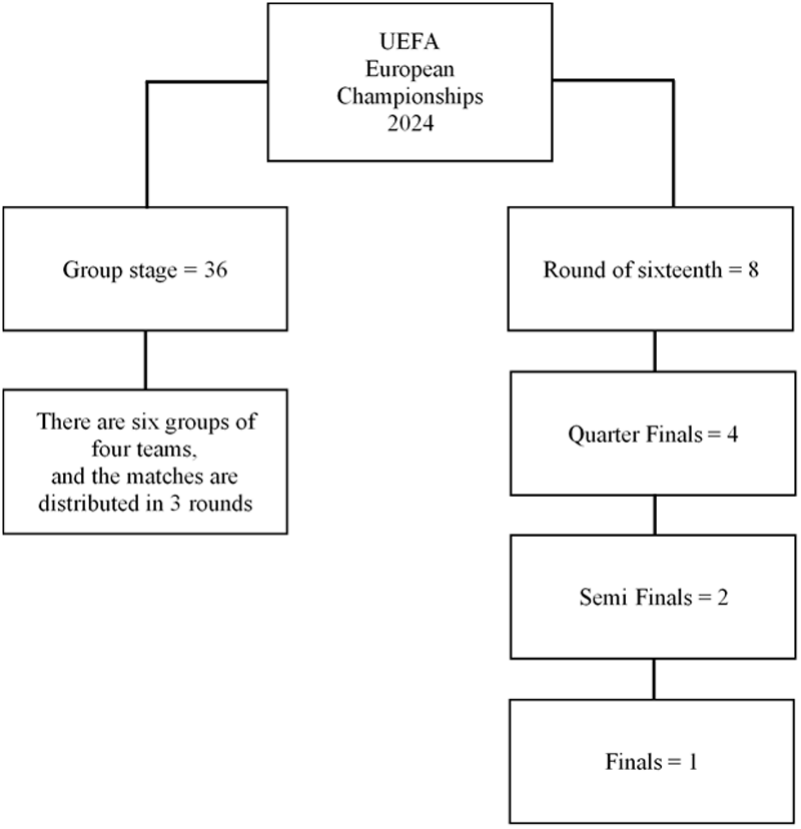
Classification of the analyzed UEFA European Championship 2024 matches.

Previous studies indicate many variables influencing coaches' decisions regarding substitutions. The UEFA European Championship 2024 is a specific high-profile sporting event, in which the best teams in Europe participate. The role of the coach of these teams is also specific during the entire preparations and sports competitions. Here, as in the case of club teams, we do not have a coach working with players for many months, systematically building the team during daily training. In the case of the national team, this work is based primarily on match observations and team composition for a selected tactical construct. This is an unusual situation for a coach working with a team. Therefore, analyzing the impact of situational variables on the time and tactics of substitutions performed by national team coaches is a further expansion of knowledge on this subject.

The analysis of the time structure of substitutions and their effectiveness based on a literature review allows us to hypothesize that substitutions made by coaches between the 60th and 85th minute of the match during UEFA EURO 2024 are significantly more probability to have a positive impact on the match outcomes compared with substitutions made before the 60th minute or after the 85th minute.

## 2 Materials and method

### 2.1 Study design

The data for the analysis were obtained from the official UEFA EURO 2024 match reports. The research aims to present the coaching decisions and their consequences based on statistical tests and a predictive machine learning (ML) component to identify relationships between the variables, time interval information, the impact of substitution, and the frequency of player substitutions in football. This study includes data analysis from individual phases, i.e., the entire tournament, the group stage, and the knockout stage.

### 2.2 Sample

All men's UEFA EURO 2024 tournament matches were analyzed (*n* = 51) (Figure 1).

### 2.3 Variables and instrument

**Figure 2:**
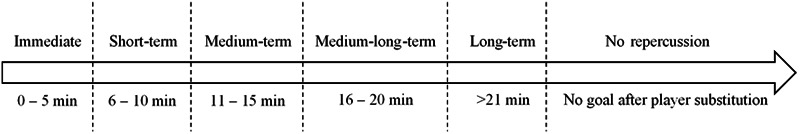
Interpretation of the RPSG variable (34).

The variables used were based on the analytical approach from Iglesias et al. (34), as well as their categories as follows:

1. Competition stage (CS): (1) group stage and (2) knockout stage.
2. Match status (MS): result at the time the coaches made any player substitution: (1) tying, (2) winning, and (3) losing.
3. Player substitution time (PST): minute in which the player substitutions were made:
  a. (0) no player substitution;
  b. (1) minute 0:00 to end of the first half;
  c. (11) minute 45:00 to 50:00;
  d. (12) minute 50:01 to 55:00;
  e. (13) minute 55:01 to 60:00;
  f. (14) minute 60:01 to 65:00;
  g. (15) minute 65:01 to 70:00;
  h. (16) minute 70:01 to 75:00;
  i. (17) minute 75:01 to 80:00;
  j. (18) minute 80:01 to 85:00;
  k. (19) minute 85:01 to 90:00;
  l. (20) minute 90:01 to end of the match.
4. Goal time (GT): minute in which a goal was scored. It presents the same categories as the PST variable.
5. Repercussion of the player substitutions on goals (RPSG): A classification was established that considers the period of time that elapsed from the player substitution until a goal was scored. This classification uses 5 min intervals (Figure 2). This variable was calculated as:
  RPSG=GT-PST
6. Impact of RPSG (IRPSG): (1) positive impact (own team goal after a player substitution), (2) negative impact (goal by the opposing team after a player substitution), and (3) no impact.
7. Final result (FR) of the match: (1) tie, (2) win, and (3) loss.

### 2.4 The chi-square automatic interaction detection method

The process starts with collecting the data and selecting the ML model that best suits the aim of the study. Algorithms learn from the dataset and are then used to predict results or assess the current situation.

The chi-square automatic interaction detection (CHAID) method was used due to its properties in the analysis of categorical variables and its ability to detect complex interactions between multiple predictors through a hierarchical structure in the context of team management and coaching decisions.

As mentioned by Milanović and Stamenković (35), the decision tree is one of the most frequently used methods for effectively segmenting data based on statistically significant relationships, which aligns with our aim to identify combinations of match status, substitution timing, and final result that are most impactful.

### 2.5 Statistical analysis

All the statistical analyses were performed with OriginPro 2024 (OriginLab Corporation, USA). Statistical significance was set at *α* ≤ 0.05. For the analysis of the degree of relationship between the variables, the chi-square test (*χ*^2^) was used, and Cramer's V coefficient (Vc) indicated the strength of association between the variables. The strength of association was assessed by Crewson's guidelines, obtained value: <0.100 (small), 0.100–0.299 (low), 0.300–0.499 (moderate), and >0.500 (high) (36). After that, the adjusted standardized residuals (ASR) from the contingency tables were examined to detect patterns of association (37). Finally, the machine learning (ML) feature was applied to create the decision trees that were used to predict and identify interactions. The CHAID method was used to achieve interaction between variables by the academic open-source program Jeffrey's Amazing Statistics Program (JASP) (38).

## 3 Results

A total of 466 substitutions in the UEFA EURO 2024 tournament were analyzed, of which 328 player substitutions occurred during the group stage and 138 substitutions during the knockout stage (Table 1). The most common substitution according to player position was midfielder to midfielder (*n* = 126) of the entire tournament and individual phases (*n* = 92 and 34, respectively). A comparison of substitutions according to player position reveals a significant offensive advantage over the defensive (74 vs. 30) of the entire tournament. Similarly, the result is divided into individual phases: the group stage (48 vs. 18) and the knockout stage (26 vs 12).

**Table 1:** Player substitutions including the player's position in the UEFA EURO 2024 tournament.

Phase	Substitute					
	Replaced	Goalkeeper	Defender	Midfielder	Forward	Total
Tournament	Goalkeeper	1	0	0	0	1
	Defender	0	53	24	17	94
	Midfielder	0	17	126	57	200
	Forward	0	13	42	116	171
	Total	1	83	192	190	466
Group stage	Goalkeeper	1	0	0	0	1
	Defender	0	38	16	9	63
	Midfielder	0	11	92	39	142
	Forward	0	7	28	87	122
	Total	1	56	136	135	328
Knockout stage	Goalkeeper	0	0	0	0	0
	Defender	0	15	8	8	31
	Midfielder	0	6	34	18	58
	Forward	0	6	14	29	49
	Total	0	27	56	55	138

There are significant associations between the RPSG, FR, and IRPSG variables of the entire tournament with a moderate and high strength of association (Table 2). In individual phases, we can also notice the same results of significant associations between variables, except for the non-significant association in the knockout stage in short-term relationships with small strength. The no-repercussion relationship is statistically insignificant and was rejected in further analysis.

**Table 2:** Degree of relationship between the RPSG, FR, and IRPSG variables of the UEFA EURO 2024 tournament.

Phase	Relationship	*χ* ^2^	df	*p*-value	Vc	Strength
Tournament	Immediate × FR × IRSPG	17.590	2	0.0001*	0.528	High
	Short-term × FR × IRSPG	9.658	2	0.007*	0.473	Moderate
	Medium-term × FR × IRSPG	17.324	2	0.0001*	0.561	High
	Medium-long-term × FR × IRSPG	15.259	2	0.0004*	0.582	High
	Long-term × FR × IRSPG	47.799	2	<0.0001*	0.633	High
	No repercussion × FR × IRSPG	0	0	1	–	–
Group stage	Immediate × FR × IRSPG	6.127	2	0.0467*	0.418	Moderate
	Short-term × FR × IRSPG	11.856	2	0.002*	0.599	High
	Medium-term × FR × IRSPG	6.474	2	0.039*	0.418	Moderate
	Medium-long-term × FR × IRSPG	11.085	2	0.003*	0.571	High
	Long-term × FR × IRSPG	26.648	2	<0.0001*	0.612	High
	No repercussion × FR × IRSPG	0	0	1	–	–
Knockout stage	Immediate × FR × IRSPG	13.739	2	0.001*	0.700	High
	Short-term × FR × IRSPG	0.104	1	0.746	0.102	Small
	Medium-term × FR × IRSPG	14.142	2	0.0008*	0.886	High
	Medium-long-term × FR × IRSPG	6.16	2	0.045*	0.748	High
	Long-term × FR × IRSPG	21.296	1	<0.0001*	0.666	High
	No repercussion × FR × IRSPG	0	0	1	–	–

*χ*^2^, chi-square test; df, degrees of freedom; Vc, Cramer's V coefficient; RSPG, repercussion of the player substitutions on goals; FR, final result; IRPSG, impact of the repercussion of the player substitutions on goals. * indicates a statistically significant difference according to conditions.

A significant result of ASRs was observed in all matches where the final result was a win or loss. Still, a non-insignificant result was observed in matches ending in a tie of the entire tournament section (Table 3). In the group stage, ASRs were significant in wins or losses, except for immediate RPSG of defeat matches. All matches ending in a tie had insignificant ASR results. A significant result of ASRs was observed in the knockout stage, in immediate, medium-term, medium-long-term, and long-term time intervals where the final result was winning and in immediate, medium-term, and long-term time intervals of lost matches. A non-insignificant result was observed in all matches where the final result was a tie.

**Table 3:** ASRs of the crossover of the RPSG, FR, and IRPSG variables of the UEFA EURO 2024 tournament.

Phase	RPSG	IRPSG	FR								
			Tie			Win			Loss		
			*n*	%	ASRs	*n*	%	ASRs	*n*	%	ASRs
Tournament	Immediate	POS	11	25.58	0.049	25	58.13	3.594*	7	16.27	−3.867*
		NEG	5	25	−0.049	2	10	−3.594*	13	65	3.867*
	Short-term	POS	5	19.23	0.130	17	65.38	2.684*	4	15.38	−2.97*
		NEG	3	17.64	−0.130	4	23.52	−2.684*	10	58.82	2.97*
	Medium-term	POS	6	26.08	1.652	12	52.17	3.195*	5	21.73	−4.142*
		NEG	3	9.37	−1.652	4	12.5	−3.195*	25	78.12	4.142*
	Medium-long-term	POS	4	17.39	−1.438	15	65.21	3.881*	4	17.39	−2.602*
		NEG	8	36.36	1.438	2	9.09	−3.881*	12	54.54	2.602*
	Long-term	POS	17	28.33	1.245	37	61.66	5.797*	6	10	−6.637*
		NEG	11	18.64	−1.245	7	11.86	−5.797*	41	69.49	6.637*
Group stage	Immediate	POS	8	33.33	−0.688	12	50	2.325*	4	16.66	−1.809
		NEG	5	45.45	0.688	1	9.09	−2.325*	5	45.45	1.809
	Short-term	POS	5	25	0.125	12	60	3.004*	3	15	−3.164*
		NEG	3	23.07	−0.125	1	7.69	−3.004*	9	69.23	3.164*
	Medium-term	POS	4	25	0.824	8	50	1.992*	4	25	−2.512*
		NEG	3	14.28	−0.824	4	19.04	−1.992*	14	66.66	2.512*
	Medium-long-term	POS	4	23.53	−0.752	11	64.70	3.176*	2	11.76	−2.566*
		NEG	6	35.29	0.752	2	11.76	−3.176*	9	52.94	2.566*
	Long-term	POS	17	45.94	1.170	18	48.64	3.797*	2	5.40	−4.862*
		NEG	11	32.35	−1.170	3	8.82	−3.797*	20	58.82	4.862*
Knockout stage	Immediate	POS	3	15.78	1.261	13	68.42	2.832*	3	15.78	−3.698*
		NEG	0	0	−1.261	1	11.11	−2.832*	8	88.88	3.698*
	Short-term	POS	N/A	N/A	N/A	5	83.33	0.322	1	16.66	−0.322
		NEG	N/A	N/A	N/A	3	75	−0.322	1	25	0.322
	Medium-term	POS	2	28.57	1.880	4	57.14	2.842*	1	14.28	−3.760*
		NEG	0	0	−1.880	0	0	−2.842*	11	100	3.760*
	Medium-long-term	POS	0	0	−1.712	4	66.66	2.288*	2	33.33	−0.884
		NEG	2	40	1.712	0	0	−2.288*	3	60	0.884
	Long-term	POS	N/A	N/A	N/A	19	82.60	4.614*	4	17.39	−4.614*
		NEG	N/A	N/A	N/A	4	16	−4.614*	21	84	4.614*

RPSG, repercussion of the player substitutions on goals; IRPSG, impact of the repercussion of the player substitutions on goals; POS, positive impact; NEG, negative impact; *n*, frequency; FR, final result; ASR, adjusted standardized residuals. * indicates statistical significance.

Regardless of the type of RPSG, the positive impact implies more cases in matches that end in a win (106 cases), while the negative impact implies more cases in matches that end in a loss (101 cases) of the entire tournament. Comparing matches that result in a tie, the positive impact (43 cases) outweighs the negative impact (30 cases). In a percentage comparison, the positive impact associated with winning the game was between 52% and 65%, while the negative impact related to losing the game was between 54% and 78%. We observe a more positive impact of players' substitutions in winning matches made in short-term or long-term time intervals. The more negative impact of player substitutions in losing matches was made in the medium-term or long-term time intervals.

In the group stage, the positive impact, regardless of the type of RPSG, implies more cases in matches that end in a win (61 cases), while the negative impact implies more cases in matches that end in a loss (57 cases). In the comparison of matches that result in ties, there is the advantage of positive impact (38 cases) than negative impact (28 cases). In a percentage comparison, the positive impact associated with winning the game was between 48% and 64%, while the negative impact related to losing the game was between 45% and 69%. We observe that the most positive impact of player substitutions in winning matches was made in the long-term time interval. The more negative impact of player substitutions in losing matches was made in the long-term time interval.

In the knockout stage, the positive impact, regardless of the type of RPSG, implies more cases in matches that end in a win (45 cases), while the negative impact implies more cases in matches that end in a loss (44 cases). In the comparison of matches that result in a tie, there is the advantage of positive impact (five cases) than negative impact (two cases). In a percentage comparison, the positive impact associated with winning the game was between 57% and 83%, while the negative impact related to losing the game was between 25% and 100%. We observe that the most positive impact of player substitutions in winning matches was made in the long-term time interval. The more negative impact of player substitutions in losing matches was made in the long-term time interval.

**Figure 3:**
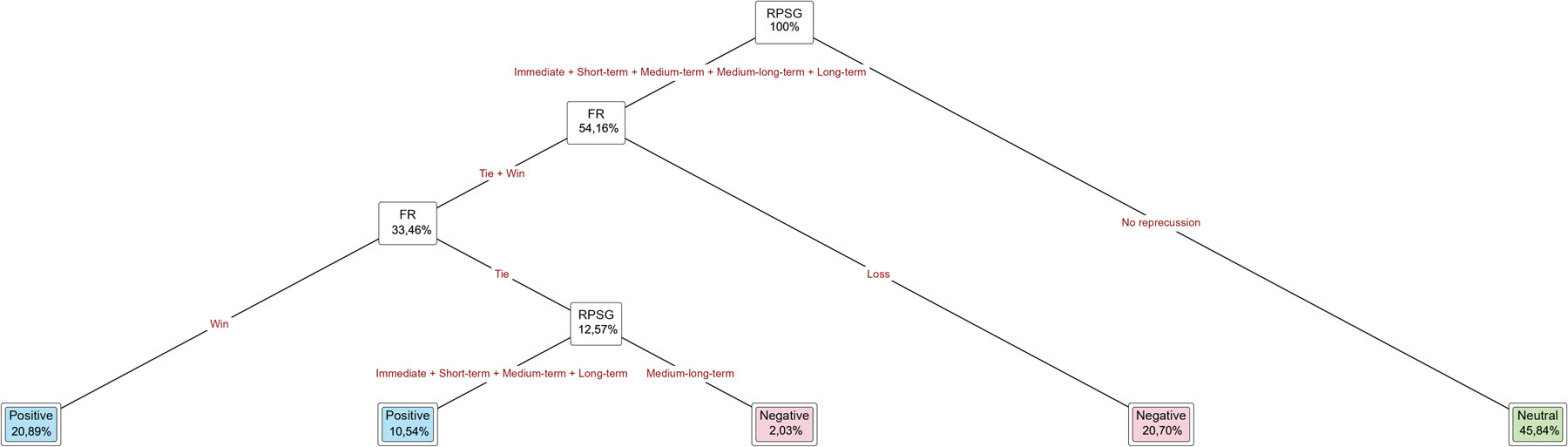
The decision tree designed with RPSG, FR as independent variable, and IRPSG as dependent variable of the entire tournament.

Figure 3 shows the decision tree designed with RPSG, FR as the independent variable, and IRPSG as the dependent variable of the entire tournament. We observe that 45.84% of the neutral impact is from no-repercussion player substitution. The positive impact is 20.89% of won matches, and the negative impact is 20.70% of lost matches. In the tie match status, the substitution of players made in the immediate, short-term, medium-term, and long-term time intervals had a 10.54% positive impact, and a 2.03% negative impact in the medium-term and long-term time intervals.

There are significant associations between all variables of the entire tournament with a moderate or high strength of association (Table 4). We can also observe significant associations between all variables in the comparison group to the knockout stage with a moderate or high strength of association.

**Table 4:** Degree of relationship between the MS, FR, and IRPSG variables of the UEFA EURO 2024 tournament.

Phase	Relationship	*χ* ^2^	df	*p*-value	Vc	Strength
Tournament	Tying × FR × IRSPG	330.299	4	<0.0001*	0.868	High
	Winning × FR × IRSPG	82.548	4	<0.0001*	0.469	Moderate
	Losing × FR × IRSPG	88.946	4	<0.0001*	0.480	Moderate
Group stage	Tying × FR × IRSPG	206.635	4	<0.0001*	0.821	High
	Winning × FR × IRSPG	73.087	4	<0.0001*	0.536	High
	Losing × FR × IRSPG	80.297	4	<0.0001*	0.553	High
Knockout stage	Tying × FR × IRSPG	121.66	4	<0.0001*	0.960	High
	Winning × FR × IRSPG	24.718	4	<0.0001*	0.453	Moderate
	Losing × FR × IRSPG	16.909	4	<0.002*	0.369	Moderate

*χ*^2^, chi-square test; df, degrees of freedom; Vc, Cramer's V coefficient; MS, match status; FR, final result; IRPSG, impact of the repercussion of the player substitutions on goals. *indicates a statistically significant difference according to conditions.

The highest number of player substitutions, with a significant result of ASRs of the entire tournament, was observed in all matches, with IRSPG having no impact where teams were tying but the final result was a tie, with IRSPG having a positive impact where teams were winning but a final result was win, and with IRSPG having a negative impact where teams are losing but final results were loss (Table 5). The highest number of player substitutions with a significant result of ASRs was observed, with IRSPG having no impact on all final results of matches in the group and knockout stages.

**Table 5:** ASRs of the crossover of the MS, FR, and IRPSG variables of the UEFA EURO 2024 tournament.

Phase	MS	IRPSG	FR								
			Tie			Win			Loss		
			*n*	%	ASRs	*n*	%	ASRs	*n*	%	ASRs
Tournament	Tying	Positive impact	11	17.18	−6.636*	51	79.68	12.501*	2	3.12	−4.679*
		Negative impact	6	10.52	−7.297*	0	0	−4.898*	51	89.47	13.377*
		No impact	97	98.97	12.509*	1	1.02	−7.112*	0	0	−7.525*
	Winning	Positive impact	3	5.76	−1.995*	49	94.23	2.644*	0	0	−1.673
		Negative impact	22	45.83	7.415*	19	39.58	−9.015*	7	14.58	4.589*
		No impact	1	1.14	−4.701*	86	98.85	5.519*	0	0	−2.515*
	Losing	Positive impact	29	49.15	8.307*	6	10.16	3.750*	24	40.67	−9.402*
		Negative impact	2	4.44	−2.423*	0	0	−1.372	43	95.55	2.865*
		No impact	0	0	−5.621*	0	0	−2.302*	89	100	6.258*
Group stage	Tying	Positive impact	9	22.5	−5.321*	29	72.5	9.804*	2	5	−3.048*
		Negative impact	6	15.78	−6.108*	0	0	−3.511*	32	84.21	10.601*
		No impact	74	98.66	9.957*	1	1.33	−5.582*	0	0	−6.483*
	Winning	Positive impact	0	0	−2.947*	30	100	3.102*	0	0	−0.792
		Negative impact	22	62.85	8.076*	11	31.42	−8.546*	2	5.71	2.311*
		No impact	1	1.61	−4.714*	61	98.38	5.002*	0	0	−1.392
	Losing	Positive impact	29	65.90	8.581*	2	4.54	2.003*	13	29.54	−8.960*
		Negative impact	0	0	−2.816*	0	0	−0.657	23	100	2.940*
		No impact	0	0	−5.964*	0	0	−1.392	64	100	6.228*
Knockout stage	Tying	Positive impact	2	8.33	−3.740*	22	91.66	7.599*	0	0	−3.904*
		Negative impact	0	0	−4.033*	0	0	−3.652*	19	100	8.124*
		No impact	23	100	7.608*	0	0	−4.201*	0	0	−3.777*
	Winning	Positive impact	3	13.63	2.335*	19	86.36	−0.052	0	0	−1.777
		Negative impact	0	0	−0.934	8	61.53	−3.011*	5	38.46	4.440*
		No impact	0	0	−1.501	25	100	2.567*	0	0	−1.973
	Losing	Positive impact	0	0	−0.812	4	26.66	3.660*	11	73.33	−2.556*
		Negative impact	2	9.09	1.938	0	0	−1.533	20	90.90	0.115
		No impact	0	0	−1.181	0	0	−1.699	25	100	2.118*

*n*, frequency; MS, match status; IRPSG, impact of the repercussion of the player substitutions on goals; FR, final result; ASR, adjusted standardized residuals. * indicates statistical significance.

**Figure 4:**
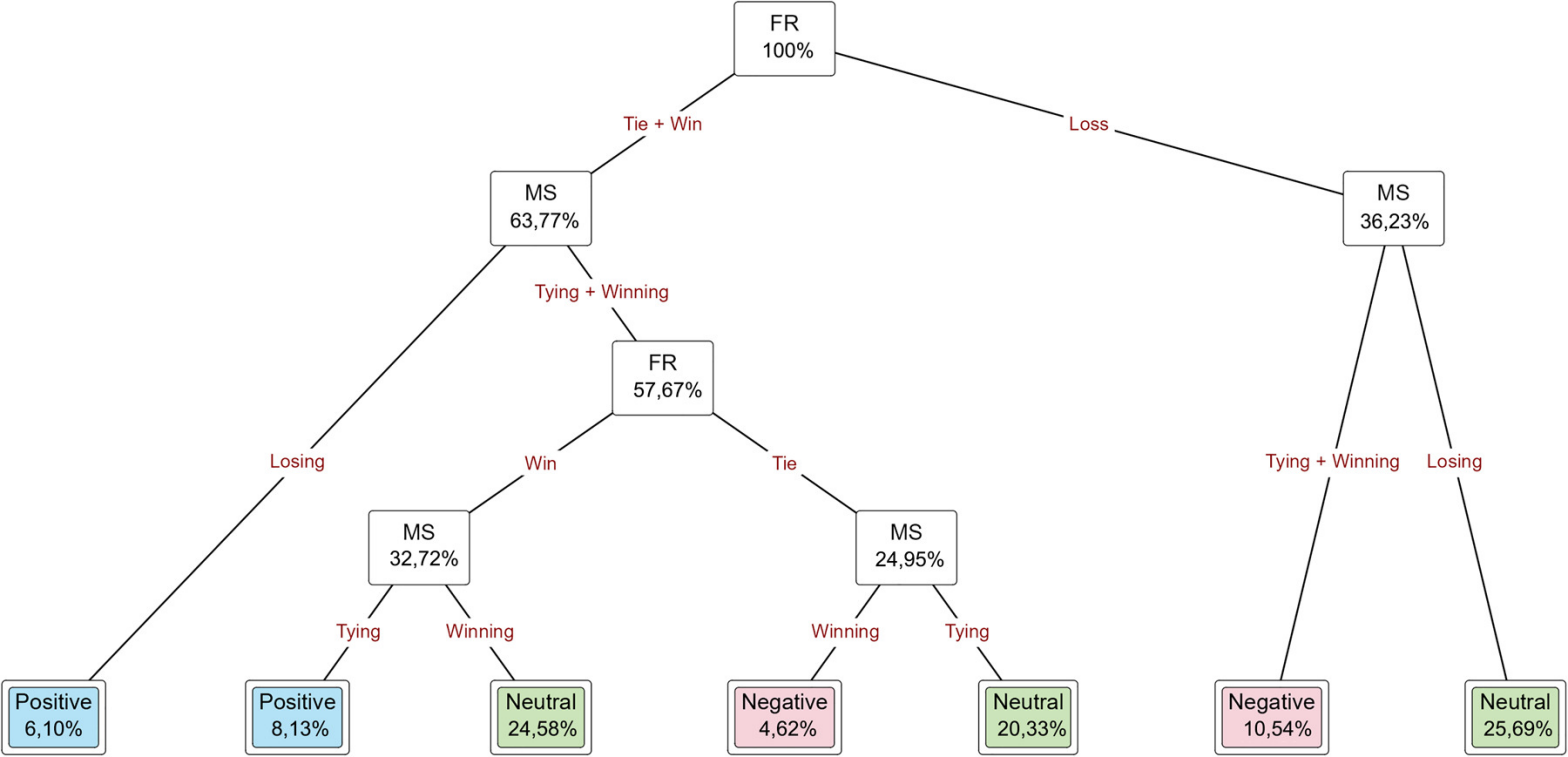
The decision tree designed with MS, FR as independent variable, and IRPSG as dependent variable of the entire tournament.

Figure 4 shows the decision tree results designed with MS, FR as the independent variable, and IRPSG as the dependent variable of the entire tournament. We observe a 6.10% positive impact of player substitutions where the match status was losing, but the final result was a win. The neutral impact is 20.33% when the match status was tying and the final result was a tie and 24.58% when the match status was winning and the final result was a win. The positive impact of tying matches, which ended with a win, is 8.13%, and the negative impact of tying or winning matches, which ended with a loss, is 10.54%. There was a 4.62% negative impact of player substitutions on winning match status, but the final results were tied.

There are significant associations between variables of the entire tournament, with matches in which the match status was tied having a moderate strength of association (Table 6). The matches in which the match status was winning or losing are statistically insignificant, but with a low strength of association. Significant associations exist between variables in the group stage, with matches in which the match status was tying or winning with a moderate strength of association. The matches in which the match status was losing are statistically insignificant, but with a moderate strength of association. There are significant associations between variables in the knockout stage, with matches in which the match status was tying with a high strength of association. The matches in which the match status was winning or losing are statistically insignificant, but with a moderate strength of association.

**Table 6:** Degree of relationship between the MS, FR, and PST variables of the UEFA EURO 2024 tournament.

Phase	Relationship	*χ* ^2^	df	*p*-value	Vc	Strength
Tournament	Tying × FR × PST	53.428	20	<0.0001*	0.349	Moderate
	Winning × FR × PST	21.675	20	0.358	0.240	Low
	Losing × FR × PST	20.849	20	0.406	0.232	Low
Group Stage	Tying × FR × PST	48.069	20	0.0004*	0.396	Moderate
	Winning × FR × PST	31.154	16	0.012*	0.350	Moderate
	Losing × FR × PST	21.951	20	0.343	0.289	Low
Knockout Stage	Tying × FR × PST	39.160	18	0.002*	0.544	High
	Winning × FR × PST	23.631	20	0.258	0.443	Moderate
	Losing × FR × PST	16.152	18	0.581	0.360	Moderate

*χ*^2^, chi-square test; df, degrees of freedom; Vc, Cramer's V coefficient; MS, match status; FR, final result; PST, player substitution time. * indicates a statistically significant difference according to conditions.

Throughout the entire tournament, when a team is tying, if a player substitution was made in 60:01–65:00 min, we observe that 40% of the matches ended in a win (Table 7). If the substitution was made before 60 min, 33%–37% of the matches ended in a tie, and 33%–66% of the matches ended in a loss. When a team is winning, if a player substitution was made between 55 and 90 min, we observed that 67%–94% of the matches ended in a win. If the substitution was made before 55 min, we observed three cases of the matches ending in a tie, and the winning team had a 6%–15% chance of the player's substitution ending in a loss. When a team is losing, if a player substitution was made from 45:00 to 55:00 min, we observed that 5%–40% of the matches ended in a tie. If the substitution was made between 45 and 90 min, we observed that 66%–94% of the matches ended in a loss. There were a few cases of player substitution, ranging from 4% to 16%, when a team that was losing made a change to the final result to win the match.

**Table 7:** ASRs of the crossover of the MS, FR, and PST variables of the entire tournament.

MS	PST	FR								
		Tie			Win			Loss		
		*n*	%	ASRs	*n*	%	ASRs	*n*	%	ASRs
Tying	0:00 to the end of the first half	0	0	−2.588*	2	33.33	0.559	4	66.66	2.462*
	Minute 45:00 to 50:00	9	33.33	−2.079*	9	33.33	1.250	9	33.33	1.183
	Minute 50:01 to 55:00	3	37.5	−0.839	0	0	−1.607	5	62.5	2.576*
	Minute 55:01 to 60:00	6	35.29	−1.440	1	5.88	−1.802	10	58.82	3.470*
	Minute 60:01 to 65:00	12	40	−1.422	12	40	2.252*	6	20	−0.578
	Minute 65:01 to 70:00	15	68.18	1.596	4	18.18	−0.646	3	13.63	−1.219
	Minute 70:01 to 75:00	15	65.21	1.335	6	26.08	0.279	2	8.69	−1.835
	Minute 75:01 to 80:00	8	47.05	−0.429	3	17.64	−0.615	6	35.29	1.111
	Minute 80:01 to 85:00	12	52.17	0.012	6	26.08	0.279	5	21.73	−0.291
	Minute 85:01 to 90:00	15	68.18	1.596	6	27.27	0.410	1	4.54	−2.269*
	90:01 to the end of the match	19	79.16	2.817*	3	12.5	−1.371	2	8.33	−1.923
Winning	0:00 to the end of the first half	0	0	−0.402	1	100	0.464	0	0	−0.197
	Minute 45:00 to 50:00	3	15	0.149	17	85	0.328	0	0	−0.933
	Minute 50:01 to 55:00	0	0	−0.571	2	100	0.658	0	0	−0.280
	Minute 55:01 to 60:00	0	0	−1.502	11	84.61	0.221	2	15.38	2.292*
	Minute 60:01 to 65:00	8	25.80	2.097*	21	67.74	−2.336*	2	6.45	0.869
	Minute 65:01 to 70:00	3	13.63	−0.038	19	86.36	0.525	0	0	−0.9847
	Minute 70:01 to 75:00	1	5.55	−1.076	17	94.44	1.415	0	0	−0.880
	Minute 75:01 to 80:00	6	24	1.567	19	76	−0.895	0	0	−1.059
	Minute 80:01 to 85:00	2	8.33	−0.844	20	83.33	0.134	2	8.33	1.268
	Minute 85:01 to 90:00	2	12.5	−0.169	14	87.5	0.564	0	0	−0.824
	90:01 to the end of the match	1	6.66	−0.844	13	86.66	0.456	1	6.66	0.621
Losing	0:00 to the end of the first half	0	0	−1.178	0	0	−0.482	7	100	1.312
	Minute 45:00 to 50:00	4	17.39	0.184	1	4.34	0.364	18	78.26	−0.333
	Minute 50:01 to 55:00	2	40	1.477	0	0	−0.405	3	60	−1.198
	Minute 55:01 to 60:00	1	5.88	−1.197	0	0	−0.773	16	94.11	1.457
	Minute 60:01 to 65:00	8	23.52	1.306	2	5.88	1.026	24	70.58	−1.671
	Minute 65:01 to 70:00	4	16.66	0.086	1	4.16	0.319	19	79.16	−0.221
	Minute 70:01 to 75:00	4	14.28	−0.276	0	0	−1.025	24	85.71	0.710
	Minute 75:01 to 80:00	2	15.38	−0.068	0	0	−0.668	11	84.61	0.359
	Minute 80:01 to 85:00	6	28.57	1.653	1	16.66	0.462	14	66.66	−1.746
	Minute 85:01 to 90:00	0	0	−1.764	0	0	−0.722	15	100	1.964*
	90:01 to the end of the match	0	0	−1.088	1	16.67	1.943*	5	83.33	0.158

MS, match status; FR, final result; PST, player substitution time; *n*, frequency; ASR, adjusted standardized residuals. * indicates statistical significance.

In the group stage, when a team is tying, if a player substitution was made in 60:01–65:00 min, we observed that 47% of the matches ended in a win (Table 8). If the substitution was made after 65 min, we observed that 64%–83% of the matches ended in a tie, and if the substitution was made before 65 min, we observed that 25%–62% of the matches ended in a loss. When a team is winning, if a player substitution was made between 60 and 90 min, we observed that 66%–93% of the matches ended in a win. If the substitution was made between 45:00 and 50:00 min, we observed three cases of the matches ending in a tie, and if the substitution was made before 60 min, we observed two cases of the matches ending in a loss. When a team is losing, if a player substitution is made after 55 min, we observed that 60%–93% of the matches ended in a loss. There was a player substitution when the losing team made a substitution and won the match.

**Table 8:** ASRs of the crossover of the MS, FR, and PST variables in the group stage.

Group stage										
MS	PST	FR								
		Tie			Win			Loss		
		*n*	%	ASRs	*n*	%	ASRs	*n*	%	ASRs
Tying	0:00 to the end of the first half	0	0	−1.678	0	0	−0.703	2	100	2.663*
	Minute 45:00 to 50:00	9	56.25	−0.164	3	18.75	−0.091	4	25	0.282
	Minute 50:01 to 55:00	3	37.5	−1.217	0	0	−1.434	5	62.5	2.814*
	Minute 55:01 to 60:00	6	40	−1.502	1	6.66	−1.329	8	53.33	3.051*
	Minute 60:01 to 65:00	9	42.85	−1.531	10	47.61	3.480*	2	9.52	−1.506
	Minute 65:01 to 70:00	14	77.77	1.795	2	11.11	−0.966	2	11.11	−1.207
	Minute 70:01 to 75:00	11	64.70	0.579	4	23.52	0.431	2	11.76	−1.100
	Minute 75:01 to 80:00	7	53.84	−0.330	1	7.69	−1.131	5	38.46	1.472
	Minute 80:01 to 85:00	12	70.58	1.100	2	11.76	−0.863	3	17.64	−0.481
	Minute 85:01 to 90:00	13	65	0.664	6	30	1.255	1	5	−1.987*
	90:01 to the end of the match	5	83.33	1.274	1	16.66	−0.185	0	0	−1.335
Winning	0:00 to the end of the first half	N/A	N/A	N/A	N/A	N/A	N/A	N/A	N/A	N/A
	Minute 45:00 to 50:00	3	18.75	0.071	13	81.25	0.100	0	0	−0.541
	Minute 50:01 to 55:00	N/A	N/A	N/A	N/A	N/A	N/A	N/A	N/A	N/A
	Minute 55:01 to 60:00	0	0	−1.549	8	80	−0.026	2	20	4.875*
	Minute 60:01 to 65:00	8	33.33	2.150*	16	66.66	−1.867	0	0	−0.688
	Minute 65:01 to 70:00	3	23.07	0.490	10	76.92	−0.324	0	0	−0.481
	Minute 70:01 to 75:00	1	6.25	−1.317	15	93.75	1.445	0	0	−0.541
	Minute 75:01 to 80:00	3	21.42	0.341	11	78.57	−0.173	0	0	−0.501
	Minute 80:01 to 85:00	2	12.5	−0.623	14	87.5	0.773	0	0	−0.541
	Minute 85:01 to 90:00	2	16.66	−0.136	10	83.33	0.276	0	0	−0.460
	90:01 to the end of the match	1	16.66	−0.094	5	83.33	0.190	0	0	−0.317
Losing	0:00 to he end of the first half	0	0	−0.934	0	0	−0.218	3	100	N/A
	Minute 45:00 to 50:00	4	28.57	0.613	0	0	−0.492	10	71.42	−0.457
	Minute 50:01 to 55:00	2	40	0.980	0	0	−0.283	3	60	−0.876
	Minute 55:01 to 60:00	1	6.66	−1.533	0	0	−0.512	14	93.33	1.645
	Minute 60:01 to 65:00	6	28.57	0.775	2	9.52	3.261*	13	61.90	−1.698
	Minute 65:01 to 70:00	4	20	−0.250	0	0	−0.604	16	80	0.418
	Minute 70:01 to 75:00	4	20	−0.250	0	0	−0.604	16	80	0.418
	Minute 75:01 to 80:00	2	28.57	0.421	0	0	−0.338	5	71.42	−0.313
	Minute 80:01 to 85:00	6	40	1.770	0	0	−0.512	9	60	−1.581
	Minute 85:01 to 90:00	0	0	−1.657	0	0	−0.387	9	100	1.730
	90:01 to the end of the match	0	0	−0.759	0	0	−0.177	2	100	0.793

MS, match status; FR, final result; PST, player substitution time; *n*, frequency; ASR, adjusted standardized residuals. * indicates statistical significance.

In the knockout stage, when a team is tying, if a player substitution was made after 70 min, we observed that 25%–77% of the matches ended in a tie (Table 9). If the substitution was made before 70 min, we observed that 22%–54% of the matches ended in a win. If the substitution was made after 80 min, we observed that 33% of the matches ended in a loss. There was a situation when the winning team made a player substitution, and the final result was a tie or loss. When a team is losing, if a player substitution was made in 60–65 min, there were cases where matches ended in a tie. If the substitution was made after 45 min, we observed that 75%–88% of the matches ended in a loss. There were a few cases in which a player substitution changed the final result to win the match.

**Table 9:** ASRs of the crossover of the MS, FR, and PST variables in the knockout stage.

Knockout stage										
MS	PST	FR								
		Tie			Win			Loss		
		*n*	%	ASRs	*n*	%	ASRs	*n*	%	ASRs
Tying	0:00 to the end of the first half	0	0	−1.611	2	50	0.729	2	50	0.966
	Minute 45:00 to 50:00	0	0	−2.837*	6	54.54	1.634	5	45.45	1.337
	Minute 50:01 to 55:00	N/A	N/A	N/A	N/A	N/A	N/A	N/A	N/A	N/A
	Minute 55:01 to 60:00	0	0	−1.121	0	0	−1.015	2	100	2.258*
	Minute 60:01 to 65:00	3	33.33	−0.302	2	22.22	−0.760	4	44.44	1.116
	Minute 65:01 to 70:00	1	25	−0.547	2	50	0.729	1	25	−0.172
	Minute 70:01 to 75:00	4	66.66	1.524	2	33.33	0	0	0	−1.633
	Minute 75:01 to 80:00	1	25	−0.547	2	50	0.729	1	25	−0.172
	Minute 80:01 to 85:00	0	0	−2.006	4	66.66	1.816	2	33.33	0.257
	Minute 85:01 to 90:00	2	100	1.839	0	0	−1.015	0	0	−0.913
	90:01 to the end of the match	14	77.77	4.091*	2	11.11	−2.345*	2	11.11	−1.942*
Winning	0:00 to the end of the first half	0	0	−0.231	1	100	0.395	0	0	−0.304
	Minute 45:00 to 50:00	0	0	−0.474	4	100	0.812	0	0	−0.624
	Minute 50:01 to 55:00	0	0	−0.329	2	100	0.564	0	0	−0.433
	Minute 55:01 to 60:00	0	0	−0.407	3	100	0.697	0	0	−0.535
	Minute 60:01 to 65:00	0	0	−0.645	5	71.42	−1.261	2	28.57	2.061*
	Minute 65:01 to 70:00	0	0	−0.746	9	100	1.276	0	0	−0.981
	Minute 70:01 to 75:00	0	0	−0.329	2	100	0.564	0	0	−0.433
	Minute 75:01 to 80:00	3	27.27	3.750*	8	72.72	−1.504	0	0	−1.106
	Minute 80:01 to 85:00	0	0	−0.697	6	75	−1.042	2	25	1.832
	Minute 85:01 to 90:00	0	0	−0.474	4	100	0.812	0	0	−0.624
	90:01 to the end of the match	0	0	−0.746	8	88.88	0.212	1	11.11	0.327
Losing	0:00 to the end of the first half	0	0	−0.377	0	0	−0.543	4	100	0.676
	Minute 45:00 to 50:00	0	0	−0.529	1	25	0.615	8	88.88	−0.157
	Minute 50:01 to 55:00	N/A	N/A	N/A	N/A	N/A	N/A	N/A	N/A	N/A
	Minute 55:01 to 60:00	0	0	−0.262	0	0	−0.377	2	100	0.470
	Minute 60:01 to 65:00	2	15.38	2.791*	0	0	−1.065	11	84.61	−0.782
	Minute 65:01 to 70:00	0	0	−0.377	1	25	1.561	3	75	−1.071
	Minute 70:01 to 75:00	0	0	−0.553	0	0	−0.795	8	100	0.992
	Minute 75:01 to 80:00	0	0	−0.470	0	0	−0.676	6	100	0.843
	Minute 80:01 to 85:00	0	0	−0.470	1	16.66	1.071	5	83.33	−0.609
	Minute 85:01 to 90:00	0	0	−0.470	0	0	−0.676	6	100	0.843
	90:01 to the end of the match	0	0	−0.377	1	25	1.561	3	75	−1.071

MS, match status; FR, final result; PST, player substitution time; *n*, frequency; ASR, adjusted standardized residuals. * indicates statistical significance.

**Figure 5:**
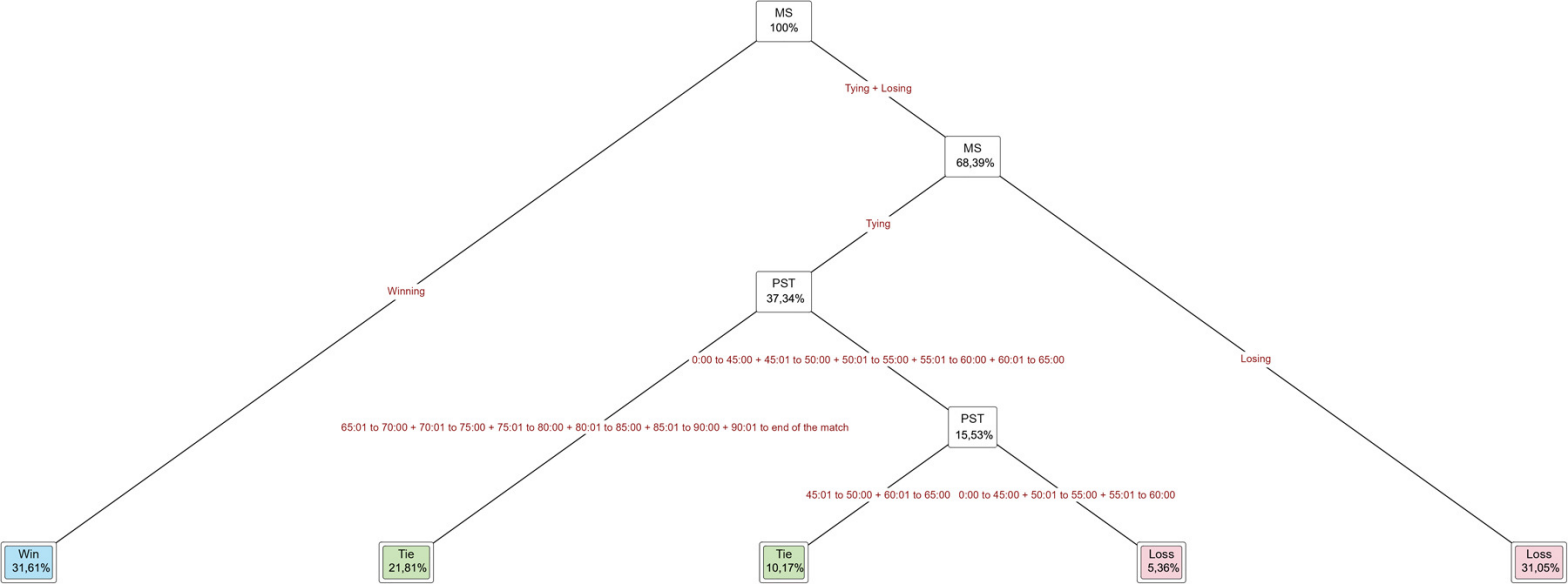
The decision tree designed with MS, PST as independent variable, and FR as dependent variable of the entire tournament.

Figure 5 shows the decision tree results designed with MS, PST as the independent variable, and FR as the dependent variable. We observe that 31.34% of all player substitutions occurred in matches where the final result was a win and 32.53% in matches where the final result was a loss. Moreover, 14.79% of substitutions from 65 min to the end of the match resulted in a tie. In a match where status was tying, 5.18% of matches with player substitution time from 0 to 60 min ended in a loss, and 16.27% of matches with player substitution time from 45 to 80 min ended in a tie.

**Figure 6:**
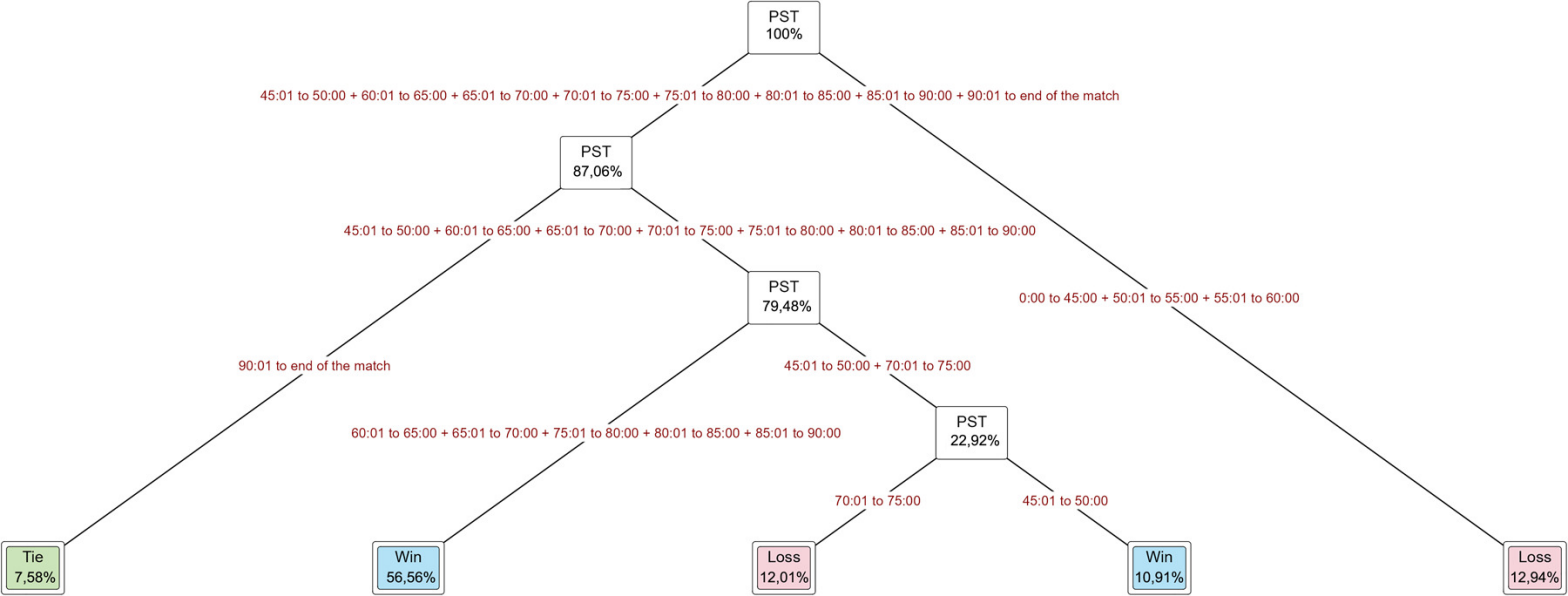
The decision tree designed with PST as independent variable and FR as dependent variable of the entire tournament.

Figure 6 shows the decision tree results designed with PST as the independent variable and FR as the dependent variable of the entire tournament. We can observe the player substitutions made from 0 to 60 min (12.94%) and from 70 to 75 min (12.01%) accounted for matches that ended in a loss and those ade from 45 to 50 min (10.91%) and from 60 to 90 min (56.56%) accounted for matches that ended in a win. In the final result of tie matches, the player substitution time from 90 min to the end of the match was at 7.58%.

## 4 Discussion

The analysis of the decisions made by coaches of the national teams participating in the UEFA 2024 tournament aimed to identify the factors determining the substitutions made in the research, which is the subject of this work. The nature of the tournament and its sports regulations practically eliminated the possibility of a draw or a controlled defeat. From the second stage, each match had to be resolved, which increased the teams' determination to win the match in regular time. Therefore, it can be assumed that the basic tactical assumption in each match was a victory, and as time passed, the determination increased among both the players and the coach. The basic assumption of the analysis was the current match result, which was assumed to be the basic determinant of decisions about the selection and substitutions of players made by coaches of the best national teams in Europe. An important element that may differentiate coaches' decisions about making substitutions may be the stage of the competition. Previous research did not consider this factor in the analysis of replacement football players. In the case of the UEFA EURO 2024 tournament, the first stage, i.e., the group stage, gave the team a chance to advance to the next round despite defeat. The knockout stage did not provide this possibility. The “all or nothing” rule forces coaches to respond decisively to the result (20). The studies of Rey et al. (19), Myers (16), and Del Corral et al. (18) indicate that the status of the match and its current score are closely related to tactical changes and substitutions made. A match that can't lose in a lead situation forces the coach to decide to strengthen the defense. In an unfavorable result, decisions are directed at taking control of the center zone of the pitch and strengthening the attack formation. In the second case, decisions about the first substitution are made earlier. In the UEFA EURO 2024 tournament, decisions about defensive substitutions in the first and second stages of the tournament were made at a rate of 2.3 and 2.7 per match, respectively. Therefore, the coaches strengthened the defense much more in the knockout stage compared with the group stage. Decisions about offensive changes were also made more frequently in the knockout stage compared with the group stage, 4.1 and 4.2 per match, respectively. The decision to strengthen the attack led to a tactical solution most often used to change the result by deciding the match or leading to a draw. Tactical changes made during the match are neutral, defensive, and offensive (18, 19, 39). It is the current match score that influences the decisions made by the coach (1, 20). Defensive substitutions increase the quality of the team's defensive formation and condense the team's defensive space. Offensive substitutions increase control of the opponent's defensive space and reduce the distance between teams. In total, decisions to strengthen defense or attack formations by substituting a player, which affected the change in the team's formation, which is important for these formations, were made in 339 cases out of 466 changes made in the entire tournament. Therefore, 72.74% of coaches’ decisions about player substitutions resulted from the decision to change the team's tactics toward more offensive (214 changes) or defensive (125 changes). In the match time structure during the UEFA EURO 2024 tournament, the time intervals in which coaches have so far most often made decisions about substitutions are 61–65 min, 71–75 min, and 81–85 min. They correspond to the time intervals of the most common substitutions determined in the studies by Bradley et al., Myers, and Rey et al. (5, 16, 19). At the same time, it has been shown that the match status has a strong impact on the decision-making time. In the knockout stage, almost twice as many substitutions as in the three typical time periods mentioned were made after the 90th minute of the match, i.e., during added time or extra time. These are tactical substitutions aimed at quickly changing the result, which at that moment may be unfavorable for both teams (tie), strengthen the defense (winning), or strengthen the attack (losing). These substitutions made by coaches are based on the current result, in which the decision-making process was highlighted in the studies of Lago, Bradley and Noakes, Myers, and Rey et al. (1, 4, 16, 19). The effectiveness of decisions made about substitutions was assessed using the IRSPG coefficient, which expresses the impact of substitution on the match result in 5 min periods, counted from the time of the substitution. Decisions made by coaches in the context of the time that passes between their decision-making and the team's reaction to the change are statistically significant in games ending with victory or defeat. Therefore, statistical analysis did not distinguish a period of the match in which the coach's decision was not statistically significant. From the point of view of the accuracy of decisions made, it is important to determine the structure of successful changes that brought the expected effect and unsuccessful changes after which a goal was lost. The results of the analysis indicate a slightly larger number of decisions that had a positive impact (*n* = 106) on the match result than negative ones (*n* = 101). Substitutions with a positive impact were effective for all time intervals counted from the time of the player substitution. The most common negative (69%) or positive (61%) impact occurred from the substitution of a player after the 20th minute.

In winning matches, substitutions made in all time intervals positively affected 52%–65% (106 cases). The negative effect had a different strength depending on the time that had passed since the change was made. Each time, the effect was statistically significant, with variable strength in individual time intervals, characterized by a waveform. The negative effect of the substitution was not temporary. Between 54% and 78% (101 cases) of the number of substitutions had a negative effect on the team that lost the match, while for the winning teams, the negative effect concerned between 9% and 23% (19 cases) of substitutions. Such a high difference between the effect of the substitution made by the coach indicates that when teams of similar high sports level compete, the correct (29.21%) or incorrect decision to substitution has a decisive impact on the final result (25.04%). In game tactics, the coach's decisions are often conditioned by the decisions made by the opposing team (16, 19). Therefore, substitutions are not made according to the 1:1 rule, i.e., replacement of players nominally playing in the same position. In the UEFA EURO 2024 tournament, there were 235 such substitutions in the group stage, which constituted 71.64% of all substitutions, while in the knockout stage, there were 104, which constituted 75.36% of all substitutions in the individual phases of the tournament. During the UEFA EURO 2024 tournament, a very common case was that the team defended a favorable result. One of the decisions made by the coach is to replace a defender or defensive midfielder with an offensive player. The incoming striker (in position no. 10) aims to attack the opponent high, making it difficult for them to play from the gate. This increases the amount of football action being moved further away from the home gate. Another solution is to strengthen the defense by replacing a midfielder or striker with a defender when the score is favorable. A favorable result for the own team forces the opponent to increase offensive actions, which are often carried out by crossing from the side sectors or half-spaces. Tactical changes also include reducing the opponent's advantage by making a “densifying” change in a specific sector of the field. The substitutions resulting from the need to increase or maintain the intensity of the game were drawn by Carling et al., Bradley and Noakes, Bradley et al., Pan et al., Hirotsu and Wright, Padrón-Cabo et al., and Lorenzo-Martínez et al. (3–5, 8, 17, 40, 41).

In the UEFA EURO 2024 tournament, in the group stage, most player changes were neutral, corresponding to the individual final results: 74 changes (24%), matches that ended in a tie; 61 changes (18.9%), matches that ended in a win (defensive); and 64 changes (19.51%), matches that ended in a loss (offensive). In the knockout stage, the most common type of substitution is neutral in matches with 23 substitutions ending in a tie, 25 substitutions ending in a win, and 25 substitutions ending in a loss. The reasons may be various, and decisions about substitution are made before the start of the match. The course of the competition indicates the optimal time for the coach to make such a decision. The most common reason is a decrease in the game's intensity, and the substitution is to maintain or increase it (5). Player injuries require immediate decision-making about substitution (12). Coaching decisions can be prepared in advance when a player enters the game with an injury and spontaneously when the injury results from participation in the game. Planned substitutions may involve changing the game's tactics and moving players on the pitch. This is an element of game quality management. Replacing a player on the pitch who is fully capable with another player in the same position in practice can be difficult or impossible. This is easier in the national team, but much more difficult in the club team. Then, other positions are rotated to maintain the quality of the team's play. This may require an additional substitution. Such a change in tactics is usually accompanied by double or triple substitutions. As noted in the works of Almeida et al. and Smith et al. (33, 42), an important effect of the substitutions is the team's mental sphere. These types of decisions are usually made by the coach in the event of a prolonged unfavorable score or a change in the score at the end of the match. In the UEFA UERO 2024 matches, 14 substitutions were made at the end of the match (last 5 min) in the group stage, which constituted 4.26% of the total substitutions, and in the knockout stage, 29% and 21%, respectively. Of these, 30 (9.14%) substitutions led to a change in the result in the group stage, and 25 (18.11%) in the knockout stage. The coach can use five players for substitutions in three breaks during the game and one additional break, which is the break between the first and second half of the match. This regulation limitation on substitution work requires management of breaks, in which, to use all players, there must be at least one double substitution. Using a double substitution during the break between the first and second half of the match gives the coach freedom to manage substitutions throughout the second half of the match. During the UEFA EURO 2024 tournament, this strategy was used 23 times in the group stage and 11 times in the knockout stage, which represented 7% and 8% of all substitutions in matches, respectively.

To verify various predictive models of making player substitutions, an efficient tool called machine learning (ML) was used (34, 43, 44). ML, using data on the time of the substitution, the result at which the substitution took place, scoring or losing a goal after the substitution, and the final result of the match, learns the relationships between them and estimates the effect of the decision made by the coach (45, 46). The use of ML was used to create decision trees, which are used when, as in the UEFA EURO 2024 tournament, there are many variants of decisions that involve risk. So far, ML has been used many times in team games research—ice hockey (47, 48), handball (49, 50), basketball (51), American football (52), and baseball (53, 54). ML has also been used in the analysis of decisions made in football (16, 55–57).

To determine the relationships of the UEFA EURO 2024 tournament match results, predictive analytical models and decision trees were used. The final result (FR) was used as a predictor (independent variable). The target (dependent) variables created three groups: (1) RPSG and IRPSG, (2) IRSPG and MS, and (3) MS and PT. Analysis of the decision tree of Group 1 showed that 46% of substitutions made during UEFA EURO 2024 had no impact on the result, and 21% were positive and contributed to the win. In 20% of cases, the team lost a goal after the substitution. Group 2 of dependent variables indicates that in 25% of cases, the substitutions made by the losing team are neutral and lead to no change in the result. In a similar percentage of cases, a substitution made by the winning team leads to another goal. In 20% of cases, substitutions made during a tie do not result in a change in the score. In 6% of cases, scoring a goal does not result in a win, and in 4% of cases, losing a goal after a substitution does not result in a loss. Group 3 dependent variables indicate that 31% of substitutions made by the winning or losing team do not influence the change of the match winner. No effect of changing the final result in the event of a tie is observed in 14% of substitutions made after the 65th minute of play. Research has shown that in the UEFA EURO 2024 tournament, nearly half (46%) of coaches' decisions to substitute did not affect the final result. An important issue for the coach, due to the effect of making a decision to substitution, is the predictable time of the potential effect of the substitution (4, 17, 18). The player reaches full effectiveness of action after a certain period of time has passed since joining the game (3, 5). During the UEFA EURO 2024 tournament, the most common period of time determining a positive or negative final result was the period above 21 min. The second significant time period that influenced the change in the final result was a positive effect between the 6th and 10th minute and a negative effect between the 11th and 15th minute. This results from the adaptation process to the intensity of the player himself and the remaining players from adapting tactics to the new position on the pitch (19). In the UEFA EURO 2024 tournament, only 8% of substitutions were effective within the first 5 min. The highest efficiency at the level of 20% is defined as medium-term after the substitution. Both the time interval and the nature of player substitutions played a significant role in the teams' efforts to win at the level of decisions made by the coach.

### 4.1 Limitations and future research

All matches of the UEFA EURO 2024 tournament were analyzed, but the relatively small number of matches (*n* = 51) limits the generalizability of the findings to other international competitions. The analysis relies solely on quantitative data, without considering the contextual or qualitative aspects of decision-making, such as individual coaching strategies, player readiness, communication during substitutions, changes in team tactics, changes in player performance during the game, and team cooperation on the pitch. The use of decision trees, while helpful in predictive modeling, may oversimplify dynamic game scenarios and cannot fully account for the non-linear nature of match outcomes.

The CHAID algorithm, while interpretable and appropriate for categorical analysis, does not include data on team formation or the reasons for forced substitutions, which may provide greater predictive accuracy. These factors should be considered when interpreting the model's results and in designing datasets for future studies.

Future research should aim to expand the dataset by including other international tournaments, domestic leagues, and women's competitions to assess whether similar patterns hold. Furthermore, the inclusion of kinematic data (e.g., total distance, high-speed running distance, and number of accelerations and decelerations) that indicate individual loadings on running performance could improve the assessment of the timing and effectiveness of substitutions in a match. Analyzing individual substitutions in the context of forced substitutions influences team tactics.

### 4.2 Study implications

The results of the study offer important insights for coaches and match analysts. The results suggest that changes made between the 60th and 85th minute are most likely to have a positive impact on match outcomes. Coaches should consider not only the running performance of players or changes in tactics but also the optimal periods for these changes to maximize their impact on results. The use of predictive models such as decision trees can support real-time decision-making tools in match management.

## 5 Conclusion

The UEFA EURO 2024 tournament is a collection of matches played in two systems—group and knockout, by national teams selected through European qualifications. The effects of decisions made by coaches during this tournament were partly different from those made in club teams' matches, in which previous analyses of this area of coaching work during the match were based. Earlier decision-making about substitutions is facilitated by the significantly higher quality of reserve players in national teams compared with club teams. The effectiveness of players' substitution toward the desired effect indicates the necessity of the coach's earlier reaction to the current result. This requires coaches to be willing to make player changes at the earlier stages of matches and adjust the initially adopted game strategy. The timing and nature of decisions made to make changes in the UEFA EURO 2024 tournament indicate that teams, after taking the lead, often decide to change their tactics and defend the result. Changing the result of a match as the game time runs out is difficult. Almost half of the changes do not affect the result. The most effective changes are those made in the medium-term time period. The least effective are those made in the last minutes of the match. Throughout the EURO 2024 tournament, effective substitutions that changed the match result toward a tie or win 5 min or less before the end of regular playing time were 17 and 20, respectively, which constituted 2.83% and 3.33% of all substitutions. Throughout the entire UEFA EURO 2024 tournament, only a few substitutions changed the result of the match from losing to victory. In almost half of the cases, coaches' decisions to substitute players have no impact on changing the match’s final result. The low effectiveness of changes toward reversing the match result indicates a limitation in the coach's ability to use this tool in conducting sports fights.

## Data Availability

The raw data supporting the conclusions of this article will be made available by the authors, without undue reservation.
